# The strategic role of Nursing in the Healthy Brazil Program to address socially determined diseases

**DOI:** 10.1590/1518-8345.0000.4692

**Published:** 2025-07-28

**Authors:** Ethel Leonor Maciel, Elton Carlos de Almeida

**Affiliations:** 1 Universidade Federal do Espírito Santo, Vitória, ES, Brazil.; 2 Ministério da Saúde, Brasília, DF, Brazil.

**Figure:**
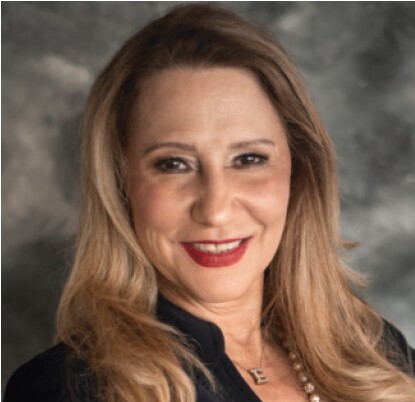

**Figure:**
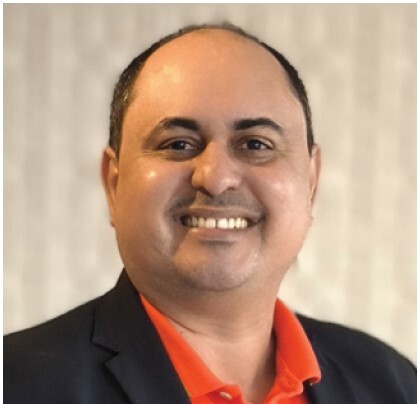

Brazilian public health is at a turning point. While neglected diseases continue to leave scars of inequality, the launch of the Healthy Brazil Program - Unite to Care (Programa Brasil Saudável - Unir para Cuidar) in 2024 emerges as an intersectoral government response, aligned with the World Health Organization (WHO) global goals, the United Nations (UN) Sustainable Development Goals (SDGs), and the Pan American Health Organization (PAHO) strategy to eliminate infections and diseases in the Americas. Coordinated by the Ministry of Health in partnership with 13 other ministries, this program aims to eliminate or reduce 11 diseases as public health concerns, including tuberculosis, leprosy, Chagas disease, and malaria - all closely linked to poverty, lack of sanitation, and territorial exclusion - along with five vertically transmitted infections ^( 1 )^.

The program is grounded in the recognition that neglected diseases are diseases of inequality and presents an opportunity to overcome health challenges by adopting a broad view of the health-disease process and its determinants. It is based on integrating technical and community knowledge and mobilizing institutional, community, public, and private resources to improve quality of life. Since the Ottawa Charter, Health Promotion has become a concept associated with values such as equity, democracy, and citizenship, involving coordinated actions among the state, community, individuals, the health system, and various sectors, reinforcing the idea of shared responsibility in seeking solutions ^( 2 )^.

In this context, it is essential to understand nursing as a strategic field with direct involvement in the development, implementation, expansion, and management of public policies in Brazil’s Unified Health System (Sistema Único de Saúde - SUS), due to its multiplier and coordinating potential across all levels of care, integrating Nursing Care Systematization into the care context.

The Ministry of Health, in partnership with the Federal Nursing Council, has been developing strategies to support the implementation of this interministerial program, considering nursing as a key player. Brazil, a continental country, requires actions that account for regional specificities. Among the 175 municipalities listed as priorities by the Healthy Brazil Program, those in the North region encompass all the diseases and infections described in the program’s National Guidelines, while municipalities in other regions focus on HIV/AIDS, tuberculosis, viral hepatitis, and syphilis ^( 3 )^.

This program sparks discussions within the context of Health Care Networks, considering Primary Health Care (Atenção Primária à Saúde - APS) as the main gateway and care coordinator, serving as a space to overcome the still predominantly disease-focused and curative care model. Health promotion actions have not yet been sufficiently consolidated to transform the approach to health production and address its determinants ^( 4 )^.

Thus, it is worth highlighting that this program represents a milestone for nursing. Nurses working on the front lines of APS are crucial in implementing strategies based on Health Promotion and Care Management, which involve linking managerial and clinical dimensions ^( 5 )^.

In this sense, nursing within the Healthy Brazil Program plays a fundamental role in developing and implementing strategies, as it can recognize local territories, identify population needs, and conduct community education to strengthen prevention, early symptom detection, and adherence to new treatments - such as the single-dose drug (tafenoquine) for malaria and the new oral drug (pretomanid) for drug-resistant tuberculosis, which reduces treatment duration from 18 to 6 months. Additionally, nursing supports intersectoral coordination with the Ministry of Cities and the Ministry of Environment and Climate Change, essential for tackling determinants like inadequate housing and deforestation.

Another critical point when discussing the Healthy Brazil Program is the coordination between SUS and the Unified Social Assistance System (Sistema Único de Assistência Social - SUAS), which seek to guarantee social rights in Brazil by promoting universal access to health and social assistance. In this context, the enhanced collaboration between nurses and social workers must be considered.

The National Health Surveillance Policy (Política Nacional de Vigilância em Saúde - PNVS) ^( 6 )^, established in 2018, reinforces the need for integration between epidemiological, sanitary, environmental, and occupational health surveillance. Healthy Brazil operates as an extension of this policy by prioritizing territorial knowledge - a principle that guides nursing to map not only pathogens but also social vulnerabilities.

An example is the use of georeferencing to identify tuberculosis risk areas, linking data from the Notifiable Diseases Information System (Sistema de Informação de Agravos de Notificação - SINAN) with migration indicators. Nurses, who lead 80% of Family Health Teams, are the agents who translate this data into local actions, such as outreach campaigns for rapid testing for syphilis, hepatitis B and C, and HIV among hard-to-reach populations like indigenous, riverside, and quilombola communities.

Despite the progress represented by Healthy Brazil, challenges persist. SUS underfunding limits access to telemonitoring technologies for chronic cases, while the precarious working conditions of nursing - with 70% of professionals overworked - threaten the program’s sustainability. However, the inclusion of nurses as prescribers of preventive treatment demonstrates unprecedented recognition of their strategic role. Medication prescription, as allowed by Brazilian Law No. 7,498 of June 25, 1986, which regulates nursing practice, enables treatment for helminthiasis, which gains new momentum under the program, prioritizing professional autonomy.

Healthy Brazil is not just another program - by linking health to human rights and environmental justice, it revives the concept of Health Surveillance as “information for action,” as defined by WHO in 1968. For nursing, this means strengthening decolonial practices, such as involving communities in research design and monitoring and advocating for investments in neglected diseases, which receive less than 1% of global health funding.

The Healthy Brazil Program is a starting point for reducing inequalities, but its success depends on transforming nursing from a “human resource” into a political actor engaged in building a health system that sees not only bodies but also territories.

## References

[1] Brasil Decreto No. 11.908, de 6 de fevereiro de 2024. Institui o Programa Brasil Saudável - Unir para Cuidar, e altera o Decreto No. 11.494, de 17 de abril de 2023, para dispor sobre o Comitê Interministerial para a Eliminação da Tuberculose e de Outras Doenças Determinadas Socialmente – CIEDDS.

[2] Buss P. M., Pellegrini A. Filho (2007). A saúde e seus determinantes sociais. Physis.

[3] Comitê Interministerial para a Eliminação da Tuberculose e de Outrad Doenças Determinadas Socialmente (2025). Diretrizes Nacionais do Programa Brasil Saudável: unir para cuidar [Internet].

[4] Malta D. C., Reis A. A. C., Jaime P. C., Morais N. O. L., Silva M. M. A., Akerman M. (2018). O SUS e a Política Nacional de Promoção da Saúde: perspectiva resultados, avanços e desafios em tempos de crise. Cien Saude Colet.

[5] Senna M. H., Drago L. C., Kirchner A. R., Santos J. L., Erdmann A. L., Andrade S. R. (2014). Meanings of care management built throughout nurses’ professional education. Rev Rene.

[6] Conselho Nacional de Saúde (BR) Resolução nº 588, de 12 de julho de 2018. Institui a Política Nacional de Vigilância em Saúde (PNVS). Diário Oficial da União [Internet].

