# Supplementary material for: The strategic role of Nursing in the Healthy Brazil Program to address socially determined diseases
Source: Rev Lat Am Enfermagem. 2025 Jul 28;33:e4692. doi: 10.1590/1518-8345.0000.4692 (PMC12306932; doi:10.1590/1518-8345.0000.4692)
Supplement: Translation

# Translation: O papel estratégico da Enfermagem no Programa Brasil Saudável para o enfrentamento das doenças determinadas socialmente

**DOI:** 10.1590/1518-8345.0000.4693

**Authors:** Ethel Leonor Maciel, Elton Carlos de Almeida

**Affiliations:** 1 Universidade Federal do Espírito Santo, Vitória, ES, Brasil.; 2 Ministério da Saúde, Brasília, DF, Brasil.

A saúde pública brasileira vive um momento de inflexão. Enquanto as doenças negligenciadas continuam a impor as cicatrizes da desigualdade, o lançamento do Programa Brasil Saudável – Unir para Cuidar, em 2024, surge como uma resposta governamental intersetorial, alinhada às metas globais da Organização Mundial da Saúde (OMS), aos Objetivos de Desenvolvimento Sustentável (ODS) da Organização das Nações Unidas (ONU) e com a estratégia da Organização Pan-Americana da Saúde (OPAS) para eliminar infecções e doenças nas Américas. Esse programa, coordenado pelo Ministério da Saúde em parceria com 13 pastas ministeriais, propõe eliminar ou reduzir como problemas de saúde pública 11 doenças, incluindo tuberculose, hanseníase, doença de Chagas e malária – todas intimamente ligadas à pobreza, à falta de saneamento e à exclusão territorial, além de cinco infecções de transmissão vertical^(1)^.

O programa parte do reconhecimento de que doenças negligenciadas são doenças da desigualdade e apresenta oportunidade de superar desafios sanitários, adotando uma visão ampla do processo saúde-doença e seus determinantes. Baseia-se na integração de saberes técnicos e populares e na mobilização de recursos institucionais, comunitários, públicos e privados para melhorar a qualidade de vida. Desde a Carta de Ottawa, a Promoção da Saúde tornou-se um conceito associado a valores como equidade, democracia e cidadania, e envolve ações coordenadas entre Estado, comunidade, indivíduos, sistema de saúde e setores diversos, reforçando a ideia de responsabilidade compartilhada na busca por soluções^(2)^. Neste contexto, faz-se necessário compreender a enfermagem como área estratégica com atuação direta na elaboração, implementação, ampliação e gestão das políticas públicas no Sistema Único de Saúde (SUS), em virtude do potencial multiplicador e articulador das equipes, em todos os níveis de atenção, integrando, no contexto do cuidado, a Sistematização de Assistência de Enfermagem.

O Ministério da Saúde, em parceria com o Conselho Federal de Enfermagem, vem construindo estratégias capazes de auxiliar no processo de implementação desse programa interministerial, considerando a enfermagem como protagonista. O Brasil, país continental, requer ações que considerem as particularidades regionais, pois, dentre os 175 municípios elencados como prioritários pelo Programa Brasil Saudável, os que estão na região Norte contemplam todas as doenças e infecções descritas nas Diretrizes Nacionais do Programa Brasil Saudável: unir para cuidar, e os municípios das demais regiões do país agrupam o HIV/Aids, Tuberculose, Hepatites Virais e Sífilis^(3)^.

A proposta desse programa suscita discussões no contexto das Redes de Atenção à Saúde, considerando a Atenção Primária à Saúde (APS) como principal porta de entrada e ordenadora do cuidado, sendo espaço para superação do modelo de atenção à saúde que continua predominantemente focado na doença e no atendimento curativo. As ações de promoção da saúde não se consolidaram suficientemente para transformar a abordagem da produção de saúde e enfrentar seus determinantes^(4)^.

Assim, vale destacar que esse programa representa um marco para a enfermagem. Enfermeiros, que atuam na linha de frente da APS, são fundamentais na implementação de estratégias fundamentadas na Promoção da Saúde e na Gestão do Cuidado que envolve a articulação entre as dimensões gerencial e assistencial^(5)^. Neste sentido, a enfermagem no contexto do Programa Brasil Saudável apresenta-se como fundamental no processo de construção e implementação de estratégias, sendo capaz de reconhecer o território e identificar as necessidades da população, além de realizar educação popular para fortalecer a prevenção, a detecção precoce de sintomas e a adesão a novos tratamentos, como, por exemplo, o novo medicamento (tafenoquina) para a malária em dose única e o novo medicamento (pretomanida), para tuberculose resistente, que passa a ser oral e reduz de 18 para 6 meses o tratamento. Além disso, subsidiar articulação intersetorial com o Ministério das Cidades e Ministério do Meio Ambiente e Mudança do Clima, essencial para combater determinantes como moradia precária e desmatamento.

Outro ponto de extrema relevância quando se pretende dialogar sobre o Programa Brasil Saudável é a articulação do SUS com o Sistema Único de Assistência Social (SUAS) que buscam garantir direitos sociais no Brasil, promovendo acesso universal à saúde e assistência social. Neste contexto, deve-se considerar a potencializada da atuação conjunta do enfermeiro com o assistente social.

A Política Nacional de Vigilância em Saúde (PNVS)^(6)^ instituída em 2018, reforça a necessidade de integração entre vigilância epidemiológica, sanitária, ambiental e do trabalhador. O Brasil Saudável opera como um desdobramento dessa política, ao priorizar o conhecimento do território – princípio que orienta a enfermagem a mapear não apenas patógenos, mas também vulnerabilidades sociais.

Exemplo disso é o uso de georreferenciamento para identificar áreas de risco para tuberculose, associando dados do Sistema de Informação de Agravos de Notificação (SINAN) a indicadores de migração. Enfermeiros, ao liderarem 80% das equipes da Saúde da Família, são os agentes que traduzem esses dados em ações locais, como mutirões para a realização de testes rápidos para Sífilis, Hepatites Virais B e C e HIV em populações de difícil acesso, como indígenas, ribeirinhas e quilombolas.

Apesar do avanço representado pelo Brasil Saudável, desafios persistem. O subfinanciamento do SUS limita o acesso às tecnologias de telemonitoramento de casos crônicos, enquanto a precarização do trabalho da enfermagem – com 70% dos profissionais sobrecarregados – ameaça a sustentabilidade do programa. No entanto, a inclusão de enfermeiros como atores da prescrição de tratamento preventivo, demonstra um reconhecimento inédito de seu papel estratégico. A prescrição de medicamentos, prevista na Lei nº 7.498, de 25 de junho de 1986 que regulamenta o exercício da enfermagem, viabiliza o tratamento para helmintíases, que ganha novo fôlego com o programa, que prioriza a autonomia profissional.

O Brasil Saudável não é apenas mais um programa – ao vincular saúde a direitos humanos e justiça ambiental, ele resgata o conceito de Vigilância em Saúde como “informação para ação”, tal como definido pela OMS, em 1968. Para a enfermagem, isso significa fortalecer práticas decoloniais, como: o envolvimento das comunidades na elaboração e acompanhamento de estudos e a defesa da necessidade de investimentos em doenças negligenciadas, que recebem menos de 1% do financiamento global em saúde.

O Programa Brasil Saudável é o ponto de partida para redução das desigualdades, mas seu sucesso depende de transformar a enfermagem de “recurso humano” a sujeito político, implicado na construção de uma saúde que enxergue, além dos corpos, os territórios.

# Translation: El papel estratégico de la Enfermería en el Programa Brasil Saludable para el enfrentamiento de enfermedades socialmente determinadas

**DOI:** 10.1590/1518-8345.0000.4691

**Authors:** Ethel Leonor Maciel, Elton Carlos de Almeida

**Affiliations:** 1 Universidade Federal do Espírito Santo, Vitória, ES, Brasil.; 2 Ministério da Saúde, Brasília, DF, Brasil.

La salud pública brasileña vive un momento de inflexión. Mientras las enfermedades desatendidas siguen dejando cicatrices de desigualdad, el lanzamiento del Programa Brasil Saludable - Unir para Cuidar (Programa Brasil Saudável - Unir para Cuidar) en 2024 surge como una respuesta gubernamental intersectorial, alineada con los objetivos globales de la Organización Mundial de la Salud (OMS), los Objetivos de Desarrollo Sostenible (ODS) de la Organización de las Naciones Unidas (ONU) y la estrategia de la Organización Panamericana de la Salud (OPS) para eliminar infecciones y enfermedades en las Américas. Coordinado por el Ministerio de Salud en colaboración con 13 carteras ministeriales, este programa busca eliminar o reducir como problemas de salud pública 11 enfermedades, incluyendo tuberculosis, lepra, enfermedad de Chagas y malaria - todas vinculadas a la pobreza, falta de saneamiento y exclusión territorial - además de cinco infecciones de transmisión vertical^(1)^.

El programa parte del reconocimiento de que las enfermedades desatendidas son enfermedades de la desigualdad y presenta una oportunidad para superar desafíos sanitarios, adoptando una visión amplia del proceso salud-enfermedad y sus determinantes. Se basa en la integración de saberes técnicos y populares y la movilización de recursos institucionales, comunitarios, públicos y privados para mejorar la calidad de vida. Desde la Carta de Ottawa, la Promoción de la Salud se ha convertido en un concepto asociado a valores como equidad, democracia y ciudadanía, e implica acciones coordinadas entre Estado, comunidad, individuos, sistema de salud y diversos sectores, reforzando la idea de responsabilidad compartida en la búsqueda de soluciones^(2)^.

En este contexto, es necesario comprender a la enfermería como un área estratégica con actuación directa en la elaboración, implementación, ampliación y gestión de políticas públicas en el Sistema Único de Salud (SUS), debido a su potencial multiplicador y articulador en todos los niveles de atención, integrando la Sistematización de la Asistencia de Enfermería en el contexto del cuidado.

El Ministerio de Salud, en colaboración con el Consejo Federal de Enfermería, ha construido estrategias para apoyar la implementación de este programa interministerial, considerando a la enfermería como protagonista. Brasil, un país continental, requiere acciones que consideren las particularidades regionales. Entre los 175 municipios priorizados por el Programa Brasil Saludable, los de la región Norte incluyen todas las enfermedades e infecciones descritas en sus Directrices Nacionales, mientras que los de otras regiones se enfocan en VIH/SIDA, tuberculosis, hepatitis virales y sífilis^(3)^.

Este programa genera discusiones en el contexto de las Redes de Atención a la Salud, considerando a la Atención Primaria de Salud (APS) como puerta de entrada principal y ordenadora del cuidado, siendo un espacio para superar el modelo aún centrado en la enfermedad y lo curativo. Las acciones de promoción de la salud no se han consolidado lo suficiente para transformar el enfoque de producción de salud y enfrentar sus determinantes^(4)^.

Así, cabe destacar que este programa representa un hito para la enfermería. Los enfermeros, que actúan en la primera línea de la APS, son fundamentales en la implementación de estrategias basadas en la Promoción de la Salud y la Gestión del Cuidado, que articulan las dimensiones gerencial y asistencial^(5)^.

En este sentido, la enfermería en el contexto del Programa Brasil Saludable es clave en la construcción e implementación de estrategias, al reconocer el territorio, identificar necesidades poblacionales y realizar educación popular para fortalecer la prevención, detección temprana de síntomas y adherencia a nuevos tratamientos - como el medicamento (tafenoquina) para malaria en dosis única y el nuevo fármaco oral (pretomanida) para tuberculosis resistente, que reduce el tratamiento de 18 a 6 meses. Además, impulsa la articulación intersectorial con el Ministerio de Ciudades y el Ministerio de Medio Ambiente y Cambio Climático, crucial para combatir determinantes como viviendas precarias y deforestación.

Otro punto relevante al dialogar sobre el Programa Brasil Saludable es la articulación entre el SUS y el Sistema Único de Asistencia Social (SUAS), que buscan garantizar derechos sociales en Brasil, promoviendo acceso universal a salud y asistencia social. Aquí, debe considerarse la potenciación del trabajo conjunto entre enfermeros y trabajadores sociales.

La Política Nacional de Vigilancia en Salud (PNVS)^(6)^, establecida en 2018, refuerza la integración entre vigilancia epidemiológica, sanitaria, ambiental y laboral. Brasil Saludable opera como un despliegue de esta política, priorizando el conocimiento del territorio - principio que guía a la enfermería a mapear no solo patógenos, sino también vulnerabilidades sociales.

Un ejemplo es el uso de georreferenciación para identificar áreas de riesgo de tuberculosis, asociando datos del Sistema de Información de Enfermedades de Notificación (Sistema de Informação de Agravos de Notificação - SINAN) con indicadores migratorios. Los enfermeros, que lideran el 80% de los equipos de Salud Familiar, traducen estos datos en acciones locales, como campañas de pruebas rápidas para sífilis, hepatitis B/C y VIH en poblaciones de difícil acceso (indígenas, ribereñas, quilombolas).

A pesar de los avances, persisten desafíos: el subfinanciamiento del SUS limita el acceso a tecnologías de telemonitoreo, y la precarización laboral de la enfermería (70% sobrecargados) amenaza la sostenibilidad del programa. Sin embargo, la inclusión de enfermeros como prescriptores de tratamiento preventivo refleja un reconocimiento inédito de su papel estratégico. La prescripción de medicamentos, prevista en la ley brasileña N° 7.498/1986, facilita el tratamiento de helmintiasis, priorizando autonomía profesional.

Brasil Saludable no es solo un programa más: al vincular salud con derechos humanos y justicia ambiental, rescata el concepto de Vigilancia en Salud como “información para la acción” (OMS, 1968). Para la enfermería, esto implica fortalecer prácticas decoloniales, como la participación comunitaria en investigaciones y la defensa de inversiones en enfermedades desatendidas (que reciben <1% del financiamiento global).

El Programa Brasil Saludable es un punto de partida para reducir desigualdades, pero su éxito depende de transformar a la enfermería de “recurso humano” a sujeto político, implicado en construir una salud que vea, más allá de los cuerpos, los territorios.
